# Self-Administered Dietary Supplements, Herbal Products, and Ergogenic Aids Before Plastic Surgery: A Narrative Review and the PLASTIC-S Perioperative Management Framework

**DOI:** 10.7759/cureus.114938

**Published:** 2026-08-21

**Authors:** Luiz Augusto Sousa Oliveira, Nélio Nunes Cabette Filho, Victor da Costa Sacksida Valladão, Ligia Helena Mendes, Fábio André Zanella

**Affiliations:** 1 General Surgery, Fundação Hospitalar Santa Terezinha de Erechim (FHSTE), Erechim, BRA; 2 Plastic Surgery, Hospital Niterói D'Or, Serviço de Pós-Graduação em Cirurgia Plástica Prof. Ronaldo Pontes, Niterói, BRA; 3 Plastic Surgery, Hospital Nossa Senhora da Conceição, Grupo Hospitalar Conceição, Porto Alegre, BRA

**Keywords:** bleeding risk, cannabidiol, dietary supplements, herbal medicine, patient safety, perioperative care, plastic surgery, preoperative assessment

## Abstract

The autonomous consumption of dietary supplements, high-dose vitamins, herbal products, ergogenic aids, and "natural" weight-loss preparations has increased substantially among candidates for plastic surgery. In Brazil, this pattern is amplified by a large market of compounded (manipulado) formulas that may contain undeclared active ingredients, by gym-oriented prohormones and selective androgen receptor modulators, and by the expanding access to cannabidiol products since the regulatory changes introduced by the Brazilian Health Regulatory Agency (ANVISA) in 2019 and 2022. These substances are frequently omitted during the preoperative interview because patients do not perceive them as medications, yet several of them have been associated, to varying degrees, with perioperative concerns such as increased bleeding, hemodynamic instability under anesthesia, cytochrome P450-mediated interactions, and impaired wound healing. This narrative review synthesizes the available evidence and proposes a plastic-surgery-specific, clinically applicable framework built on two elements: the PLASTIC-S structured anamnesis mnemonic, which organizes self-administered substances into eight categories mapped to perioperative risk mechanisms, and a two-dimensional Procedure × Supplement Risk Matrix, which translates that assessment into individualized, graduated suspension intervals rather than a single flat recommendation. Procedures are stratified into four risk tiers and supplements into four risk tiers, whose intersection yields suggested suspension intervals ranging from none to about three weeks, together with a group of substances that are best avoided altogether. The framework is intended to move everyday practice beyond the generic "suspend two weeks before" advice toward a more individualized and reproducible approach, and it can be incorporated as a low-cost component of the preoperative safety routine in contemporary Brazilian plastic surgery. As a narrative review, its recommendations rest largely on pharmacological reasoning, case reports, and consensus rather than on prospectively validated evidence, and the PLASTIC-S framework should therefore be regarded as a proposed clinical tool requiring prospective validation.

## Introduction and background

The use of dietary supplements, high-dose vitamins, herbal products, ergogenic aids, and "natural" weight-loss preparations has increased substantially over the past two decades [1,2]. In Brazil, this growth is amplified by several particular features: a large and loosely regulated market of compounded (manipulado) formulas that may contain undeclared active ingredients such as sibutramine, thyroid hormones, or anorexigens [3]; a fitness culture in which preworkout stimulants, prohormones, and selective androgen receptor modulators (SARMs) are available through retail and informal channels [4,5]; the widespread use of such over-the-counter ergogenic products [6]; and a marked expansion in access to cannabidiol (CBD) and other cannabis-derived products following resolutions RDC 327/2019 and RDC 660/2022 issued by the Brazilian Health Regulatory Agency (ANVISA) [7,8].

Candidates for plastic surgery represent a population of particular interest. Most procedures are elective, and the physician-patient relationship is built on the expectation of safe and aesthetically rewarding outcomes, yet these same patients are frequently heavy consumers of wellness products, predominantly women of productive age with high exposure to marketing from the wellness, fitness, and aesthetic industries [9-11]. In everyday practice, they may take fish oil for the joints, vitamin E for the skin, ginkgo for memory, compounded weight-loss formulas in the weeks preceding mammaplasty or abdominoplasty, preworkout supplements that continue until close to the day of surgery, melatonin or CBD oil for sleep, and "detox" teas for body-contouring optimization, often continuing these products up to the perioperative period [11].

The cardinal clinical problem is that little of this appears in the routine anamnesis. When asked whether they take any medication, many patients answer no, because within their own conceptual framework these substances are food, vitamins, or natural products rather than medications [12,13]. The omission is cultural rather than intentional, but its potential consequences are concrete and include increased intraoperative bleeding, postoperative hematoma, ecchymosis, fat-graft loss, hemodynamic instability under general anesthesia, prolonged sedation, and impaired wound healing [14-16].

The literature on perioperative supplements in plastic surgery has been shaped since 2001 by the review of Ang-Lee et al. [14], followed by the practical review of Broughton et al. in 2007 [15] and by a series of updates focused on bleeding risk and perioperative drug interactions [17-20]. These works established the foundational knowledge but share two limitations from the standpoint of contemporary Brazilian practice: they are organized by substance rather than by clinical decision, leaving the practitioner with a long list of agents and a flat "suspend two weeks before" recommendation, and they do not contemplate the particular regulatory and cultural landscape in which manipulados, gym-grade prohormones, clandestine sibutramine and postregulation CBD are everyday realities [17,18].

This study aims to translate the available evidence into a clinically applicable, plastic-surgery-specific framework that addresses the current gap in practical, context-adapted guidance. Two elements are proposed: the PLASTIC-S structured anamnesis mnemonic, designed to systematize disclosure of self-administered substances by categories that map to perioperative risk mechanisms, and a two-dimensional Procedure × Supplement Risk Matrix, which translates the categorization into individualized suspension intervals stratified by procedure-specific risk, in keeping with contemporary perioperative guidance on the management of dietary supplements before surgery [21].

## Review

Search strategy and study selection

This narrative review was structured according to the domains of the Scale for the Assessment of Narrative Review Articles (SANRA), which were used to guide the synthesis and reporting [22]. We searched PubMed/MEDLINE, Scopus, Latin American and Caribbean Health Sciences Literature (Literatura Latino-Americana e do Caribe em Ciências da Saúde) (LILACS) and Scientific Electronic Library Online (SciELO) for records published between 2000 and 2026, with the last search performed in January 2026. The search combined controlled and free-text descriptors "herbal supplements," "dietary supplements," "nutraceuticals," "perioperative," "preoperative," "plastic surgery," "aesthetic surgery," "bleeding," "cytochrome P450," "wound healing," "cannabinoids," "CBD," "selective androgen receptor modulators," and "manipulados" in English, Portuguese, and Spanish, using the Boolean operators AND and OR (e.g., in PubMed: (“dietary supplements” OR “herbal supplements” OR “nutraceuticals”) AND (“perioperative” OR “preoperative” OR “plastic surgery” OR “aesthetic surgery”) AND (“bleeding” OR “cytochrome P450” OR “wound healing”)). Reference lists of key articles were hand-searched, and society guidelines and Brazilian regulatory documents (ANVISA) were consulted [7,8,21].

Sources were eligible when they addressed the pharmacology, perioperative safety, or clinical management of self-administered supplements, herbal products, ergogenic aids, or cannabinoids relevant to elective surgery, with priority given to human studies and sources of direct perioperative relevance. We excluded records without a retrievable abstract, those unrelated to the perioperative or pharmacological questions addressed here, and publications outside the three search languages. Titles and abstracts were screened for relevance, and potentially relevant full texts were then assessed against these criteria. Where evidence was graded, randomized controlled trials and meta-analyses were prioritized, followed by pharmacological reviews, society guidelines, and case reports of severe perioperative complications, given that randomized trials are ethically impracticable for many of these agents; animal or in vitro data are identified as such in the text. Consistent with its narrative (SANRA) design, this review did not apply a formal per-database record count, a Preferred Reporting Items for Systematic Reviews and Meta-Analyses (PRISMA) flow diagram, or a quantitative risk-of-bias/GRADE assessment; the categorization that follows is operational and intended for clinical applicability rather than as a strict pharmacological taxonomy [23].

The PLASTIC-S framework for perioperative-relevant supplements

**Figure 1:**
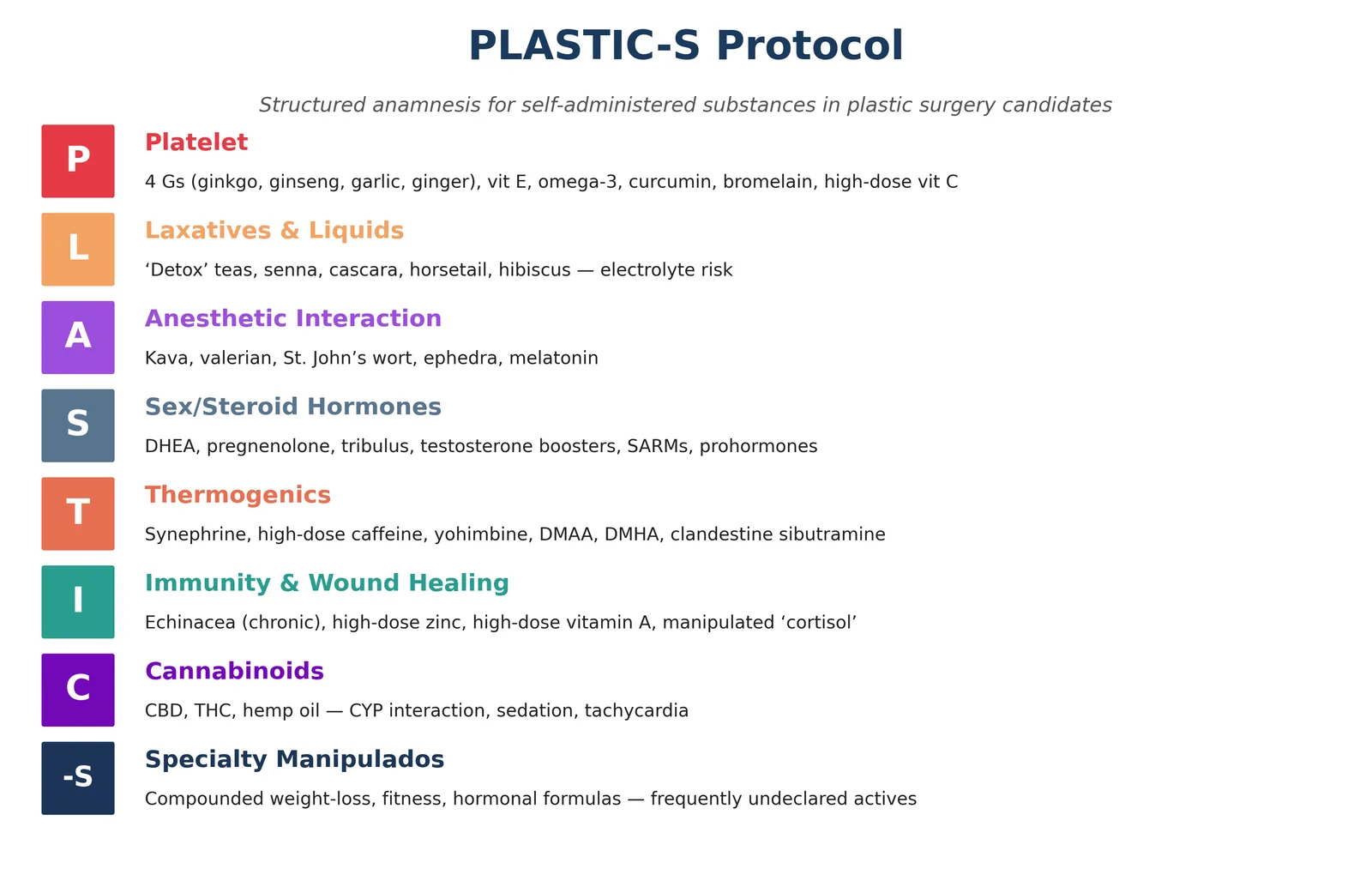
PLASTIC-S protocol-structured anamnesis mnemonic for self-administered substances in candidates for plastic surgery. Each letter represents a category mapped to a perioperative risk mechanism, prompting exemplifying questions by the surgeon Image credit: This figure was created by the authors using Python 3 with the Matplotlib library (v3.9.4)

We present the PLASTIC-S mnemonic as a proposed clinical heuristic, not a validated screening instrument, to organize the heterogeneous universe of self-administered substances into eight categories that map to perioperative risk mechanisms (Figure 1). The mnemonic is intended to be practical rather than exhaustive pharmacologically: each letter represents a category of products that patients can be asked about during a structured interview [11]. Its performance requires prospective validation before it can be regarded as a screening tool.

Platelet and Hemorrhagic Risk (P)

This is one of the most clinically relevant categories in plastic surgery. The classical mnemonic of the "4 Gs" (ginkgo, ginseng, garlic, and ginger) summarizes the most frequently cited offenders, although several other agents share the same mechanisms. *Ginkgo biloba *inhibits platelet-activating factor and has been linked in case reports to spontaneous intracranial hemorrhage, so a suspension of approximately 14 days before surgery is commonly recommended [14,24]. Panax ginseng inhibits platelet aggregation and may interact unpredictably with warfarin, for which a suspension of about seven days is generally advised; garlic (*Allium sativum)*, through ajoene, irreversibly inhibits platelet aggregation, and ginger (*Zingiber officinale*) inhibits thromboxane synthetase, with intervals of roughly one to two weeks frequently suggested [15,16].

Other agents in this category include feverfew, dong quai, high-dose curcumin, saw palmetto, horse chestnut, and bromelain. Among vitamins and fatty acids, vitamin E at doses above about 400 IU/day may exhibit an antiplatelet effect, while omega-3 fatty acids (eicosapentaenoic acid and docosahexaenoic acid (EPA/DHA)) have traditionally been thought to prolong bleeding time; the contemporary evidence is not uniform, however, and a secondary analysis of a large randomized trial found no association between fish-oil supplementation and increased perioperative bleeding, so that an individualized decision rather than routine suspension appears reasonable [17,18,25]. Vitamin C at high doses and flaxseed, evening-primrose, and borage oils have also been implicated, although the supporting evidence is comparatively weak [19].

Laxatives and Liquids (L)

"Detox" teas and slimming infusions that are widely commercialized in Brazil frequently contain undeclared stimulant laxatives, such as senna and cascara sagrada, and diuretics, such as horsetail and hibiscus at excessive doses. Their use may produce hypokalemia, hyponatremia, and dehydration in the immediate preoperative period, with potential implications for anesthetic conduction and cardiac rhythm, so a suspension of at least seven days before surgery is a reasonable precaution [5].

Anesthetic Interaction (A)

Kava-kava potentiates GABAergic central depressants and is hepatotoxic [14,26]. Valerian acts on the same receptor system and may produce benzodiazepine-like withdrawal on abrupt discontinuation, so gradual weaning is advisable, whereas St. John's wort (*Hypericum perforatum*) is a potent inducer of CYP3A4 and P-glycoprotein and alters the metabolism of midazolam, fentanyl, lidocaine, warfarin, and oral contraceptives, for which a suspension of about five days before surgery, with a warning regarding contraceptive failure, is generally recommended [27,28]. Melatonin produces additive sedation, and ephedra (ma huang), although banned by the US Food and Drug Administration in 2004, persists in clandestine formulas and has been associated with severe hypertension, tachyarrhythmia, and perioperative myocardial infarction [29,30].

Sex and Steroid Hormones (S)

Dehydroepiandrosterone (DHEA), pregnenolone,* Tribulus terrestris*, and "testosterone boosters" may modify the sex-hormone axis and have been discussed in relation to thrombotic risk [6]. A particularly relevant subgroup is that of SARMs and "prohormones" sold in fitness retail and informal markets; these compounds are frequently hepatotoxic, may suppress the hypothalamic-pituitary-gonadal axis and induce dyslipidemia, and have been associated with venous thromboembolism, so their use warrants active investigation and, when identified, medical evaluation and a suspension of at least 30 days [31]. Because these agents intersect with perioperative thrombotic risk, their identification is relevant to venous thromboembolism prophylaxis planning in body-contouring and combined procedures [32].

Thermogenics (T)

This category is particularly relevant in candidates for body-contouring procedures. Synephrine (bitter orange), high-dose caffeine, yohimbine, and the amphetamine-like compounds 1,3-dimethylamylamine (DMAA) and 1,5-dimethylhexylamine (DMHA) may cause hypertension, tachyarrhythmia, and perioperative myocardial infarction [33,34]. Clandestine sibutramine, frequently identified in manipulated formulas marketed as "natural," carries a recognized cardiovascular risk, and illicitly sold thyroid-hormone preparations may produce iatrogenic thyrotoxicosis [4,5].

Immunity and Wound Healing (I)

Chronic use of high-dose licorice, or of manipulated preparations occasionally containing hidden corticosteroids, may impair wound healing and induce immunosuppression [5]. Chronic high-dose zinc supplementation can paradoxically induce copper deficiency, anemia, and neuropathy, while echinacea in chronic use has immunomodulatory effects that have been associated with impaired healing [35,36]. High-dose vitamin A (above about 25,000 IU/day) and high-dose green-tea extract are hepatotoxic and may interfere with healing despite their physiological roles [37]. Conversely, whereas indiscriminate high-dose self-supplementation may impair recovery, structured and targeted perioperative protein and micronutrient optimization has an evidence-based role in supporting wound healing in plastic surgery, which underscores that the clinical problem lies in unsupervised excess rather than in nutrition itself [38].

Cannabinoids (C)

Following ANVISA Resolutions RDC 327/2019 and RDC 660/2022, legal access to CBD products in Brazil has expanded considerably [7,8]. Patients use cannabidiol for anxiety, sleep, chronic pain, and dysmenorrhea and may not regard it as a medication; CBD and tetrahydrocannabinol (THC) interact with cytochrome P450 isoenzymes (3A4, 2C9, 2C19) and can alter the metabolism of propofol, midazolam, warfarin, and lidocaine, while THC additionally produces tachycardia and increases anesthetic requirements [39,40]. The 2023 consensus of the American Society of Regional Anesthesia and Pain Medicine (ASRA) recommends documentation, suspension of at least 72 hours before surgery, and gradual tapering in chronic heavy users to prevent a withdrawal syndrome [41].

Specialty Manipulados (-S)

This category is specific to the Brazilian context. Compounded weight-loss formulas, fitness manipulados, and hormonal preparations sold through compounding pharmacies have been found in regulatory investigations to contain undeclared active ingredients, including sibutramine, fluoxetine, anorexigens, diuretics, thyroid-hormone fractions, and clandestine corticosteroids [4,5]. Any patient declaring the use of a compounded "weight-loss," "anxiety," or "gym" formula should be asked to provide the actual prescription, and a conservative approach is to suspend all such formulas for at least 14 days before surgery, regardless of the declared composition [3].

Procedure stratification and the risk matrix

To translate the supplement-side categorization into individualized recommendations, we propose a four-tier stratification of plastic-surgery procedures based on three perioperative risk dimensions: bleeding risk, related to vascularity and the extent of dissection; wound-healing risk, related to flap extension, tension, and patient factors such as smoking; and anesthetic risk, related to duration and complexity [15]. Tier P-I (minimal) comprises office-based procedures without dissection, such as botulinum toxin and isolated filler injections, scar revisions, and small office surgeries; tier P-II (intermediate) includes mammaplasty, blepharoplasty, isolated liposuction up to approximately three liters of aspirate, and minor secondary body-contouring procedures; tier P-III (high) includes facelift and deep-plane rhytidectomy, abdominoplasty, isolated liposuction above three liters, gluteal fat grafting, and combined breast procedures; and tier P-IV (major) includes body lift, combined major procedures, and secondary surgery after major weight loss and tier-III procedures in smokers or patients with significant comorbidities [16].

**Figure 2:**
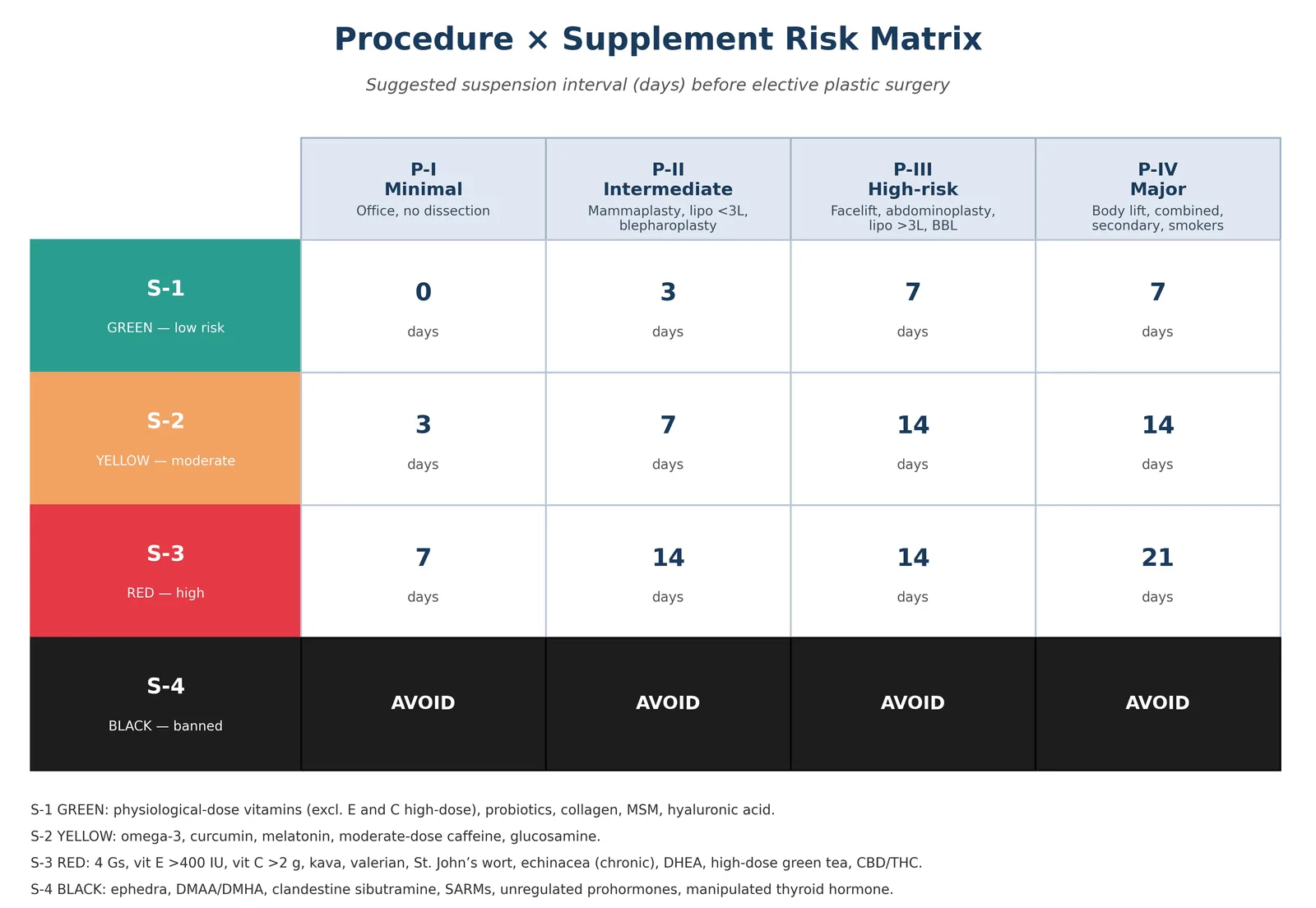
Procedure × Supplement Risk Matrix. The intersection of the supplement risk tier (S-1 green, low; S-2 yellow, moderate; S-3 red, high; S-4 black, best avoided) with the procedure risk tier (P-I to P-IV) yields the suggested suspension interval, in days, before elective plastic surgery Image credit: This figure was created by the authors using Python 3 with the Matplotlib library (v3.9.4), integrating the procedure-based and supplement-based perioperative risk stratifications proposed in the present article

Figure 2 presents the two-dimensional Procedure × Supplement Risk Matrix, a central contribution of this article. The horizontal axis represents procedure tiers (P-I to P-IV) and the vertical axis represents supplement tiers (S-1 green, S-2 yellow, S-3 red, and S-4 black), with each cell indicating a suggested suspension interval, in days, before elective surgery [14].

The supplement tiers are defined operationally. Tier S-1 (green) includes physiological-dose vitamins, excluding high-dose vitamin E and C, together with probiotics, hydrolyzed collagen, methylsulfonylmethane and hyaluronic acid, without documented perioperative interference; tier S-2 (yellow) includes omega-3, curcumin, melatonin, moderate-dose caffeine and glucosamine, with modest but documented perioperative effects; tier S-3 (red) includes the "4 Gs," high-dose vitamin E and C, kava, valerian, St. John's wort, chronic echinacea, DHEA, high-dose green-tea extract, and CBD/THC; and tier S-4 (black) comprises ephedra, DMAA/DMHA, clandestine sibutramine, SARMs, unregulated prohormones, and manipulated thyroid hormone, which are best avoided regardless of the procedure [17].

The cells of the matrix encode a graduated logic. For tier S-1 substances, no suspension is required for minimal procedures and only a brief precautionary interval of about three to seven days for higher-tier procedures; for tier S-2 substances, the suggested suspension scales from around three days for tier P-I to about 14 days for tiers P-III and P-IV; for tier S-3 substances, a conservative interval of about 14 days applies for tiers P-II and P-III, extended to about 21 days for tier P-IV; and for tier S-4 substances, avoidance with prior medical evaluation is advised irrespective of the procedure [18].

Operationalizing the framework

**Figure 3:**
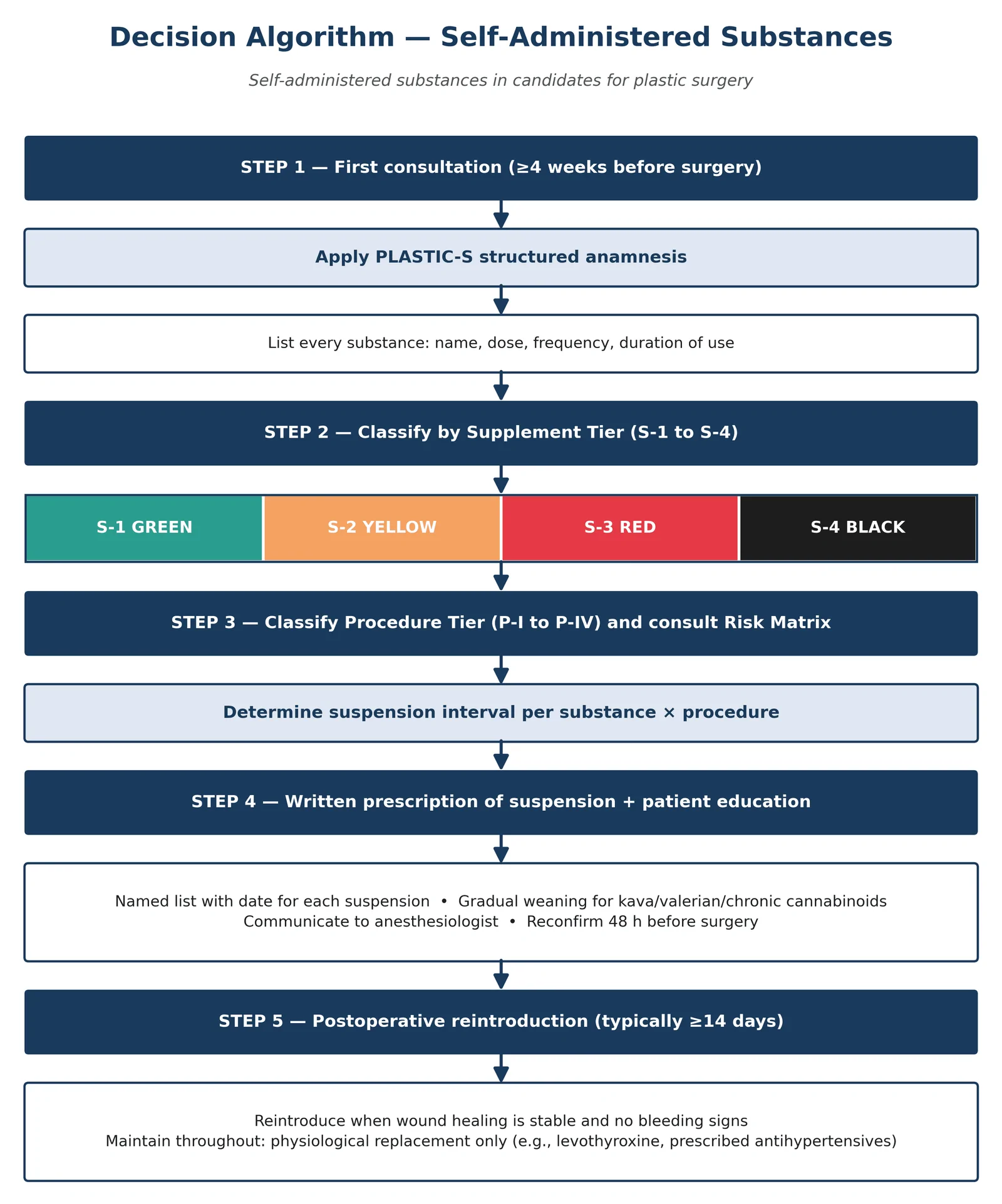
Five-step decision algorithm for self-administered substances in candidates for plastic surgery, integrating the PLASTIC-S structured anamnesis, supplement-tier and procedure-tier classification, consultation of the risk matrix, written prescription of the suspension, communication with the anesthesia team, preoperative reconfirmation, and postoperative reintroduction Image credit: This figure was created by the authors using Python 3 with the Matplotlib library (v3.9.4), integrating the PLASTIC-S structured anamnesis and the Procedure × Supplement Risk Matrix proposed in the present article

Figure 3 presents a five-step decision algorithm that operationalizes the PLASTIC-S anamnesis and the risk matrix within routine practice. It is designed to fit a typical workflow in which the first consultation occurs four or more weeks before surgery, allowing adequate time for supplement weaning; the steps comprise structured anamnesis, supplement-tier and procedure-tier classification, consultation of the matrix, written prescription of the suspension, explicit communication with the anesthesia team, preoperative reconfirmation and postoperative reintroduction (Table 1) [41].

**Table 1:** Main self-administered supplements of perioperative relevance, with PLASTIC-S category, mechanism of action, and commonly recommended suspension interval before elective plastic surgery EPA/DHA: eicosapentaenoic acid and docosahexaenoic acid; DMAA: 1,3-dimethylamylamine; DMHA: 1,5-dimethylhexylamine; CBD: cannabidiol; THC: tetrahydrocannabinol; DHEA: dehydroepiandrosterone; FDA: Food and Drug Administration Note: This table was compiled by the authors from the sources cited in the text

Supplement	PLASTIC-S category	Mechanism	Suspension
Ginkgo biloba	P-Platelet	Platelet-activating factor inhibition	~14 days
Panax ginseng	P-Platelet	Antiplatelet; hypoglycemia; warfarin interaction	~7 days
Garlic (*Allium sativum*)	P-Platelet	Irreversible antiplatelet (ajoene)	~7-10 days
Ginger (*Zingiber officinale*)	P-Platelet	Inhibits thromboxane synthetase	~14 days
Feverfew (*Tanacetum parthenium*)	P-Platelet	Antiplatelet	~7-14 days
Dong quai (*Angelica sinensis*)	P-Platelet	Coumarins	~14 days
Curcumin/turmeric (high dose)	P-Platelet	Dose-dependent antiplatelet	~14 days
Saw palmetto	P-Platelet	Antiplatelet	~14 days
Horse chestnut (*Aesculus*)	P-Platelet	Esculin (coumarin)	~14 days
Bromelain	P-Platelet	Fibrinolytic	~7-14 days
Vitamin E (>400 IU/day)	P-Platelet	Antiplatelet	~7-14 days
Omega-3 (EPA/DHA, fish/krill oil)	P-Platelet	Prolonged bleeding time (evidence not uniform)	Individualized (~7-14 days)
Flaxseed/primrose/borage oil	P-Platelet	Antiplatelet	~7-14 days
Vitamin C (>2 g/day)	P-Platelet	Possible hemostatic interference	~7 days
"Detox" teas (senna, cascara, horsetail, hibiscus)	L-Laxatives/liquids	Hidden laxatives/diuretics; hypokalemia	~7 days
Kava-kava (*Piper methysticum*)	A-Anesthetic	GABAergic potentiation; hepatotoxic	Suspend (ideally days before)
Valerian	A-Anesthetic	GABAergic; benzodiazepine-like withdrawal	Gradual weaning
St. John's wort (*Hypericum perforatum*)	A-Anesthetic	CYP3A4 and P-glycoprotein inducer	~5 days
Melatonin	A-Anesthetic	Additive sedation	Day before
Ephedra (ma huang)	A-Anesthetic (CV)	Sympathomimetic; FDA-banned	Avoid
Licorice	I-Immunity/wound healing	Hidden corticosteroids; impaired healing	~14 days
DHEA, pregnenolone, tribulus	S-Sex/steroid hormones	Hormonal disruption; thrombotic risk	~14 days
SARMs and prohormones	S-Sex/steroid hormones	Androgenic; hepatotoxic; thrombotic	~30 days + evaluation
Vitamin A (>25,000 IU/day)	I-Immunity/wound healing	Hepatotoxicity; impaired healing	~14 days
Green-tea extract (EGCG)	I-Immunity/wound healing	Hepatotoxicity at high dose	~14 days
Synephrine (bitter orange)	T-Thermogenic	Sympathomimetic	~7 days
Yohimbine, DMAA, DMHA	T-Thermogenic	Severe sympathomimetic	Avoid
Clandestine sibutramine (in manipulados)	T/-S-Manipulado	Serotonergic; cardiovascular risk	~30 days
Echinacea (chronic use)	I-Immunity/healing	Chronic immunomodulation	Suspend; avoid chronic use
Chronic high-dose zinc	I-Healing	Copper deficiency	Adjust to RDA
CBD/THC/hemp oil	C-Cannabinoids	CYP modulation; sedation; tachycardia (THC)	>=72 h; taper if chronic
Weight-loss/fitness/hormonal manipulados	-S-Specialty manipulados	Undeclared active ingredients	>=14 days

Beyond the suspension intervals summarized above, several of these substances also interact with drugs that are routinely used in the perioperative period; Table 2 details the principal supplement-drug interactions of anesthetic and hemostatic relevance, together with the affected agents, the underlying mechanism and the resulting clinical consequence [14,19,20].

**Table 2:** Clinically relevant interactions between self-administered supplements and drugs commonly used in the perioperative period, with the affected agents, the mechanism of interaction and the clinical consequence. EPA/DHA: eicosapentaenoic acid and docosahexaenoic acid; DMAA: 1,3-dimethylamylamine; DMHA: 1,5-dimethylhexylamine; CBD: cannabidiol; THC: tetrahydrocannabinol; DHEA: dehydroepiandrosterone; SARMs: selective androgen receptor modulators; SSRIs: selective serotonin reuptake inhibitors; MAOIs: monoamine oxidase inhibitors; NSAIDs: nonsteroidal anti-inflammatory drug; INR: international normalized ratio Note: This table was compiled by the authors from the sources cited in the text

Substance (PLASTIC-S category)	Perioperative agent(s) affected	Mechanism of interaction	Clinical consequence	Ref.
St. John's wort (A)	Midazolam, fentanyl, lidocaine, warfarin, oral contraceptives	Potent CYP3A4 and P-glycoprotein induction	Reduced efficacy of anesthetics and analgesics; unpredictable reduction in INR; risk of contraceptive failure; delayed emergence reported	[27,28]
CBD/THC-cannabinoids (C)	Propofol, midazolam, warfarin, lidocaine	Inhibition of CYP3A4, 2C9 and 2C19; THC sympathetic stimulation	Potentiated sedation; increased INR; THC-related tachycardia and higher anesthetic requirement; withdrawal syndrome in chronic heavy users	[39-41]
Kava-kava (A)	Benzodiazepines, propofol, volatile agents; hepatotoxic drugs	GABAergic potentiation; hepatotoxicity	Additive and prolonged CNS depression; risk of hepatotoxicity	[14,26]
Valerian (A)	Benzodiazepines, sedative-hypnotic anesthetics	GABAergic modulation	Additive sedation; abrupt discontinuation may precipitate a benzodiazepine-like withdrawal (gradual taper advised)	[14]
Melatonin (A)	Sedative-hypnotics, general anesthetics	Additive central sedation	Enhanced sedation and potential prolonged emergence	[20]
Ginkgo biloba (P)	Warfarin, antiplatelet agents, heparin, NSAIDs	Platelet-activating factor inhibition	Increased bleeding; spontaneous and intracranial hemorrhage reported	[14,24]
Panax ginseng (P)	Warfarin, antiplatelet agents, insulin/oral hypoglycemics	Antiplatelet effect; reduced warfarin effect; hypoglycemia	Variable INR; increased bleeding; hypoglycemia	[15,19]
Garlic, ginger, feverfew, dong quai (P)	Warfarin, antiplatelet agents	Antiplatelet mechanisms (ajoene, thromboxane-synthetase inhibition, coumarins)	Additive perioperative bleeding risk	[15,16,19]
High-dose vitamin E; omega-3 (EPA/DHA) (P)	Antiplatelet agents, anticoagulants	Antiplatelet effect (evidence for fish oil not uniform)	Possible additive bleeding; individualized decision reasonable for fish oil	[17,18,25]
Ephedra/ma huang (A, cardiovascular)	Volatile anesthetics, vasopressors, MAOIs	Sympathomimetic catecholamine release	Hypertension, tachyarrhythmia, perioperative myocardial infarction; intraoperative hemodynamic instability	[29,30]
Clandestine sibutramine in manipulados (T/-S)	Serotonergic agents (tramadol, fentanyl, SSRIs, MAOIs); sympathomimetics	Serotonin-noradrenaline reuptake inhibition	Hypertension, tachycardia and serotonin-syndrome risk	[4,5]
SARMs, prohormones, DHEA (S)	Warfarin; venous thromboembolism prophylaxis planning	Androgenic and procoagulant effects; hepatotoxicity	Increased thrombotic risk; possible potentiation of warfarin; hepatotoxicity	[31,32]
Licorice (I)	Antihypertensives, digoxin, diuretics	Pseudohyperaldosteronism (11-beta-hydroxysteroid dehydrogenase inhibition); occasional hidden corticosteroids	Hypokalemia (predisposing to digoxin toxicity and arrhythmia), hypertension, impaired wound healing	[5]
High-dose green-tea extract (I)	Warfarin; other hepatotoxic drugs	Dose-dependent hepatotoxicity; vitamin K content	Hepatotoxicity; potential alteration of anticoagulation	[37]
Echinacea, chronic use (I)	Immunosuppressants; CYP3A4 substrates	Chronic immunomodulation; mild CYP modulation	Impaired wound healing; theoretical pharmacokinetic interactions	[36]

Discussion

The evidence reviewed converges on several messages of practical relevance. First, the omission of supplements during the preoperative interview appears to be the rule rather than the exception and depends substantially on how the question is framed [12,13]. In interpreting the recommendations that follow, three levels should be distinguished: what is established by direct clinical evidence (e.g., the antiplatelet effect of high-dose fish oil or garlic), what is suspected on mechanistic or pharmacokinetic grounds but not yet demonstrated in surgical outcomes (e.g., several herb-anesthetic interactions), and what is proposed by this framework as a practical, expert-derived synthesis (the risk tiers and suspension intervals). The suspension intervals in particular are pharmacokinetically informed proposals rather than prospectively validated thresholds. The PLASTIC-S mnemonic addresses this by translating an unstructured list of agents into a categorical framework that prompts specific, exemplifying questions; instead of the unproductive "are you taking any medication?", the surgeon can ask about vitamins, teas, herbal products, gym supplements, shakes, drops, compounded formulas, slimming products, sleep aids and other natural products. Framing the question around recognizable product categories is more likely to elicit disclosure and can be incorporated into the institutional preprinted anamnesis form [15].

Second, the conventional flat-list "suspend two weeks before" recommendation, although operationally simple, may be suboptimal in two directions. It can overburden patients with unnecessary suspensions of low-risk supplements before low-risk procedures, which may reduce adherence and trust, and it may underestimate the interval required for high-risk substances before high-risk procedures. The Procedure × Supplement Risk Matrix aims to address both extremes by individualizing the suspension to the clinical context [17].

Third, the Brazilian context warrants explicit attention to three local realities that are not usually contemplated in international reviews: the manipulado market, the fitness market of prohormones and SARMs, and the post-RDC 327 cannabinoid market [3,6,8]. Each of these requires specific, named questioning: a patient using a compounded weight-loss formula should be asked to bring the actual prescription; a male candidate with a fitness-oriented profile should be asked specifically about prohormones and SARMs; and any patient should be asked specifically about CBD oil, regardless of how it was obtained [41].

Fourth, screening cannot be delegated entirely to the anesthesiologist, who typically meets the patient once and shortly before surgery; the plastic surgeon, often the professional with the longest-standing contact with the patient, is well-positioned to detect, document, and prescribe the suspension, and a reconfirmation about 48 hours before surgery, communicated to the anesthesia team, helps to close this loop [21]. More broadly, supplement-related bleeding and cardiovascular effects are best understood as modifiable perioperative factors that act alongside others; inadvertent perioperative hypothermia, e.g., independently impairs platelet function and coagulation, so that supplement management, thromboprophylaxis, and the maintenance of normothermia can be seen as parts of a single perioperative-safety strategy in medium- and large-volume plastic surgery [32,42].

Limitations

Several limitations should be acknowledged. Most of the evidence supporting the suspension intervals derives from case reports, pharmacological reasoning, and small case series rather than from randomized controlled trials, which are ethically unfeasible for many of these agents, so the intervals proposed in the matrix reflect pharmacokinetic estimates and prevailing consensus rather than prospectively validated thresholds [14,24]. The supplement market is highly dynamic and clandestine formulations frequently contain undeclared ingredients, so the categories presented here are illustrative rather than exhaustive and should be updated periodically [5]. In addition, the doses, formulations and actual composition of these products vary widely and are frequently undeclared, which limits the precision and generalizability of any fixed categorization or suspension interval. Methodologically, this is a narrative synthesis: it did not employ a formal protocol, a quantitative risk-of-bias assessment or a structured evidence-grading system such as GRADE, and the selection of sources therefore carries a risk of selection and interpretation bias. Finally, the PLASTIC-S mnemonic is a clinical heuristic rather than a validated screening instrument, and its impact on disclosure rates and on clinical outcomes deserves formal prospective evaluation; the heterogeneity of the underlying evidence, which spans the pharmacology of creatine, the pharmacokinetics of melatonin, broad reviews of herb-drug interactions and population data on the autonomous use of these substances in Brazil, illustrates both the breadth of the field and the caution with which its recommendations should be interpreted [43-46]. An earlier and more concise version of this framework, in Portuguese, was presented in the proceedings of a Brazilian medical congress; the present article substantially expands that preliminary work by adding the Procedure × Supplement Risk Matrix, the five-step decision algorithm and the perioperative drug-interaction table, and by situating the framework within the international literature [47].

## Conclusions

Active and explicit screening for nonprescription supplements is advisable as a routine component of the preoperative evaluation in plastic surgery. The proposed PLASTIC-S structured anamnesis and the Procedure × Supplement Risk Matrix together offer a clinically applicable, plastic-surgery-specific framework that moves practice beyond generic flatlist recommendations toward a more individualized and reproducible approach. Adapted to the Brazilian regulatory and cultural context, encompassing manipulados, gym-grade ergogenic aids, and post-RDC 327 cannabinoids, it represents a simple and low-cost intervention that may contribute to reducing perioperative complications, and it is consistent with prevailing national safety recommendations. It should be emphasized, however, that PLASTIC-S and the Procedure × Supplement Risk Matrix are presented as a preliminary clinical proposal that requires prospective validation, rather than as an established perioperative management standard; until such validation is available, they are best used as a structured aid to clinical judgment rather than a substitute for it.
